# Supplementary material for: Global Situation of Bioremediation of Leachate-Contaminated Soils by Treatment with Microorganisms: A Systematic Review
Source: Microorganisms. 2023 Mar 28;11(4):857. doi: 10.3390/microorganisms11040857 (PMC10145224; doi:10.3390/microorganisms11040857)
Supplement: Supplementary file 1 [file microorganisms-11-00857-s001.zip › microorganisms-2231004-supplementary.pdf]

Review

Global Situation of Bioremediation of Leachate-Contaminated Soils by Treatment with Microorganisms: A Systematic Review

Yesenia Mendoza-Burguete [1], María de la Luz Pérez-Rea [1], J Ledesma-García[1], Juan Campos-Guillén[1], MA Ramos-López[1], C. Guzmán[1], JA Rodríguez-Morales [1,*]

[1] Universidad Autónoma de Querétaro, Facultad de Ingeniería, Posgrado en Ingeniería, Querétaro, Qro., México

* Correspondence: jose.alberto.rodriguez@uaq.mx; Tel.: +52 4422376916

Table S1. Studies selected for the systematic review

Number	Year	Title	Reference
1	2017	Bioremediation of heavy metals from contaminated sites using potential species: a review	[1]
2	2017	A preliminary report of indigenous fungal isolates from contaminated municipal solid waste site in India	[2]
3	2017	Microbial and plant-assisted bioremediation of heavy metal polluted environments: a review	[3]
4	2017	Potential of Cordyline sp plant for remediation of metal-leachate contaminated soil	[4]
5	2017	Optimal Removal of Heavy Metals From Leachate Contaminated Soil Using Bioaugmentation Process	[5]
6	2017	Effect of inoculation with white-rot fungi and fungal consortium on the composting efficiency of municipal solid waste	[6]
7	2017	An innovative multistage treatment system for sanitary landfill leachate depuration: Studies at pilot-scale	[7]
8	2017	Assessing the bioaugmentation potentials of individual isolates from landfill on metal-polluted soil	[8]
9	2017	Characterization of induced metal responses of bacteria isolates from active non-sanitary landfill in Malaysia	[9]
10	2017	Impact of methane concentration and temperature on the activity of a methanotrophic strain isolated from a municipal landfill	[10]
11	2017	Sustainable remediation of heavy metal polluted soil: A biotechnical interaction with selected bacteria species	[11]
12	2017	A comparison of technologies for remediation of heavy metal contaminated soils	[12]

13	2017	Enhanced dissipation of DEHP in soil and simultaneously reduced bioaccumulation of DEHP in vegetable using bioaugmentation with exogenous bacteria	[13]
14	2017	Advanced technologies for the remediation of pesticide-contaminated soils	[14]
15	2017	Assessing the efficacy over time of the addition of industrial by-products to remediate contaminated soils at a pilot-plant scale	[15]
16	2017	Optimal reduction of chemical oxygen demand and NH(3)-N from landfill leachate using a strongly resistant novel Bacillus salmalaya strain..	[16]
17	2018	Application of bioremediation in the decontamination of polluted soil with petroleum hydrocarbons	[17]
18	2018	Application of biochar to sewage sludge reduces toxicity and improve organisms growth in sewage sludge-amended soil in long term field experiment	[18]
19	2018	Phytoremediation of seleniferous soil leachate using the aquatic plants Lemna minor and Egeria densa	[19]
20	2018	Characterization of Leachate and Groundwater in and Around Saduperi Municipal Solid Waste Open Dump Site, Vellore District, Tamil Nadu, India	[20]
21	2018	Bioremediation of petroleum-contaminated soil using aged refuse from landfills	[21]
22	2018	The National Program for the Liquidation of Pesticide Waste Landfills, Successes and Unused Opportunities–Case Study from Poland	[22]
23	2018	BIOTRANSFORMATION AND REMOVAL OF HEAVY METALS: A REVIEW OF PHYTO AND MICROBIAL REMEDIATION ASSESSMENT ON CONTAMINATED SOIL	[23]
24	2018	Screening of Silver-Tolerant Bacteria from a Major Philippine Landfill as Potential Bioremediation Agents	[24]
25	2018	Raoultella and Enterococcus species identified as high chromium and arsenic tolerant bacteria	[25]
26	2018	Phycoremediation of landfill leachate with the chlorophyte Chlamydomonas sp. SW15aRL and evaluation of toxicity pre and post treatment	[26]
27	2018	Metagenomics profiling for assessing microbial diversity in both active and closed landfills	[27]
28	2018	Biodegradation of dissolved humic substances by fungi	[28]
29	2018	Microbial functional diversity plays an important role in the degradation of polyhydroxybutyrate (PHB) in soil.	[29]
30	2018	Effective use of iron-aluminum rich laterite based soil mixture for treatment of landfill leachate.	[30]
31	2018	Phytoremediation of sewage sludge contaminated by trace elements and organic compounds.	[31]

32	2018	Next-generation sequencing showing potential leachate influence on bacterial communities around a landfill in China.	[32]
33	2018	Biocompatible metal decontamination from soil using Ageratum conyzoides.	[33]
34	2018	Role of Microbes in Bioremediation of Pollutants (Hydrocarbon) in Contaminated Soil	[34]
35	2018	Biomineralisation performance of bacteria isolated from a landfill in China	[35]
36	2019	Emerging sustainable technologies for remediation of soils and groundwater in a municipal solid waste landfill site – A review	[36]
37	2019	Design and start-up of a constructed wetland as tertiary treatment for landfill leachates.	[37]
38	2019	Enhanced degradation of 1-naphthol in landfill leachate using Arthrobacter sp.	[38]
39	2019	Effect of biochar on the nutrient contents and metal recovery efficiency in sorghum planted on landfill soils	[39]
40	2019	Effect of soil types and ammonia concentrations on the contribution of ammonia-oxidizing bacteria to CH4 oxidation	[40]
41	2019	Leachates and natural organic matter. A review of their biotreatment using fungi	[41]
42	2019	Can biochar and designer biochar be used to remediate per- and polyfluorinated alkyl substances (PFAS) and lead and antimony contaminated soils?	[42]
43	2019	Optimizing bioremediation of hydrocarbon polluted soil by life cycle assessment (LCA) approach.	[43]
44	2019	Effect of biostimulation on the distribution and composition of the microbial community of a polycyclic aromatic hydrocarbon-contaminated landfill soil during bioremediation	[44]
45	2019	Selective bacterial colonization processes on polyethylene waste samples in an abandoned landfill site	[45]
46	2019	Bioaccumulation and biosorption of zinc by a novel Streptomyces K11 strain isolated from highly alkaline aluminium brown mud disposal site.	[46]
47	2019	Isolation of arsenic accumulating bacteria from garbage leachates for possible application in bioremediation	[47]
48	2019	Succession and diversity of microbial communities in landfills with depths and ages and its association with dissolved organic matter and heavy metals.	[48]
49	2019	Phytoremediatory efficiency of Chrysopogon zizanioides in the treatment of landfill leachate: a case study.	[49]
50	2019	Biodegradation of polyacrylic and polyester polyurethane coatings by enriched microbial communities.	[50]

51	2019	Phytoremediation of Heavy Metal-Contaminated Soil by Switchgrass: A Comparative Study Utilizing Different Composts and Coir Fiber on Pollution Remediation, Plant Productivity, and Nutrient Leaching.	[51]
52	2019	Metabolism of diethyl phthalate (DEP) and identification of degradation intermediates by Pseudomonas sp. DNE-S1.	[52]
53	2019	Isolation of a polyethylene degrading Paenibacillus sp. from a landfill in Brazil	[53]
54	2019	A PAH-degrading bacterial community enriched with contaminated agricultural soil and its utility for microbial bioremediation	[54]
55	2019	Phytoremediation: Climate change resilience and sustainability assessment at a coastal brownfield redevelopment.	[55]
56	2019	Enhanced Bioremediation of Heavy Metal Contaminated Landfill Soil Using Filamentous Fungi Consortia: a Demonstration of Bioaugmentation Potential	[56]
57	2019	In Situ Bioremediation of Textile Dye Effluent-Contaminated Soils Using Mixed Microbial Culture	[57]
58	2019	Zooremediation of leachates from municipal waste using Eisenia fetida (SAV.).	[58]
59	2019	Transport, retention, and release of Escherichia coli and Rhodococcus erythropolis through dry natural soils as affected by water repellency.	[59]
60	2019	Biodegradation of pyrene by Pseudomonas sp. ISTPY2 isolated from landfill soil: Process optimisation using Box-Behnken design model	[60]
61	2020	Enhanced Immobilization and Phytoremediation of Heavy Metals in Landfill Contaminated Soils	[61]
62	2020	Antagonic and plant growth-promoting effects of bacteria isolated from mine tailings at El Fraile, Mexico	[62]
63	2020	A Review of Landfill Microbiology and Ecology: A Call for Modernization With ‘Next Generation’ Technology	[63]
64	2020	Microbial Degradation and Valorization of Plastic Wastes	[64]
65	2020	In-situ biodegradation of volatile organic compounds in landfill by sewage sludge modified waste-char	[65]
66	2020	Optimized bioleaching of copper by indigenous cyanogenic bacteria isolated from the landfill of e-waste	[66]
67	2020	Biotransformation of chromium (VI) by Bacillus sp. isolated from chromate contaminated landfill site	[67]
68	2020	The effect of different levels of leachate on phytoremediation of pyrene-contaminated soil and simultaneous extraction of lead and cadmium	[68]
69	2020	Improvement of soil properties and plant responses by compost generated from biomass of phytoremediation plant	[69]

70	2020	Investigation of the influence of biofertilizer synthesized using microbial inoculums on the growth performance of two agricultural crops	[70]
71	2020	Assessment of biodegradation efficiency of polychlorinated biphenyls (PCBs) and petroleum hydrocarbons (TPH) in soil using three individual bacterial strains and their mixed culture	[71]
72	2020	Evaluation of a biosurfactant producing bacterial strain Pseudomonas sp. ISTPY2 for efficient pyrene degradation and landfill soil bioremediation through soil microcosm and proteomic studies	[72]
73	2020	Soil bioremediation: Overview of technologies and trends	[73]
74	2020	Bioremediation of landfill leachate by Aspergillus flavus in submerged culture: Evaluation of the process efficiency by physicochemical methods and 3D fluorescence spectroscopy	[74]
75	2020	Effective bioremediation of heavy metal–contaminated landfill soil through bioaugmentation using consortia of fungi	[75]
76	2020	Discovery of a novel native bacterium of Providencia sp. with high biosorption and oxidation ability of manganese for bioleaching of heavy metal contaminated soils.	[76]
77	2020	Bioprospecting potential of microbial communities in solid waste landfills for novel enzymes through metagenomic approach	[77]
78	2020	In-situ biodegradation of harmful pollutants in landfill by sludge modified biochar used as biocover.	[78]
79	2020	Exploration of strategies to increase the nitrogen and phosphate content of solid waste landfill soil	[79]
80	2020	Phytoremediation of waste dumping site soil and landfill leachate by using cattail (Typha latifolia).	[80]
81	2020	Degradation of di-(2-ethylhexyl) phthalate by Bacillus aquimaris isolated from Ajakanga municipal solid waste leachate	[81]
82	2020	Bioaugmentation assisted mycoremediation of heavy metal and/metalloid landfill contaminated soil using consortia of filamentous fungi	[82]
83	2020	Screening and selection of autochthonous fungi from leachate contaminated-soil for bioremediation of different types of leachate	[83]
84	2020	Biodegradation of the plastics PLA and PET in cultivated soil with the participation of microorganisms and plants	[84]
85	2020	Evolution properties and dechlorination capacities of particulate organic matter from a landfill.	[85]
86	2021	Bioremediation of organic contaminants based on biowaste composting practices	[86]
87	2021	Effect of biochar addition on the improvement of the quality parameters of compost used for land reclamation	[87]

88	2021	Treatment of landfill leachate with different techniques: an overview	[88]
89	2021	Microbial consortia for industrial waste bioremediation: an insight to related patents	[89]
90	2021	A survey of intact low-density polyethylene film biodegradation by terrestrial Actinobacterial species	[90]
91	2021	Screening of Fungal Microbiome to Identify Potential Polyethylene Degrading Fungi	[91]
92	2021	Bioremediation of petroleum contaminated soils-A review	[92]
93	2021	Biochar derived from pig manure with ability to reduce the availability of Pb in contaminated agricultural soils	[93]
94	2021	Phytoremediation of PAHs in Contaminated Soils: A Review	[94]
95	2021	Phytoremediation of soil treated with metalliferous leachate from an abandoned industrial site by Alternanthera sessilis and Ipomoea aquatica: Metal extraction and biochemical responses	[95]
96	2021	Environmental risk assessment in selected dumpsites in Abakaliki metropolis, Ebonyi state, southeastern Nigeria	[96]
97	2021	Effect of green waste and lime amendments on biostabilisation, physical-chemical and microbial properties of the composted fine fraction of residual municipal solid waste	[97]
98	2021	Bioremediation of heavy metals and other toxic substances by microorganisms	[98]
99	2021	Aerobic composting remediation of petroleum hydrocarbon-contaminated soil. Current and future perspectives	[99]
100	2021	Potential environmental and human health risks caused by antibiotic-resistant bacteria (ARB), antibiotic resistance genes (ARGs) and emerging contaminants (ECs) from municipal solid waste (MSW) landfill	[100]
101	2021	Low density polyethylene degradation by filamentous fungi	[101]
102	2021	Actinobacteria: An eco-friendly and promising technology for the bioaugmentation of contaminants	[102]
103	2021	Feasibility Study of the Soil Remediation Technologies in the Natural Environment	[103]
104	2021	Bioremediation of clay with high oil content and biological response after restoration	[104]
105	2021	Aerobic degradation of decabrominated diphenyl ether through a novel bacterium isolated from municipal waste dumping site: Identification, degradation and metabolic pathway	[105]
106	2021	Biodegradability of di-(2-ethylhexyl) phthalate by a newly isolated bacterium Achromobacter sp. RX	[106]

107	2021	Global soil pollution by toxic elements: Current status and future perspectives on the risk assessment and remediation strategies–A review	[107]
108	2021	Relationship between Leachate Pollution Index and growth response of two willow and poplar hybrids: Implications for phyto-treatment applications	[108]
109	2021	A combined approach to remediate cadmium contaminated sediment using the acidophilic sulfur-oxidizing bacterial SV5 and untreated coffee ground	[109]
110	2021	Resistance of bacteria isolated from leachate to heavy metals and the removal of Hg by Pseudomonas aeruginosa strain FZ-2 at different salinity levels in a batch biosorption system	[110]
111	2021	Application of in situ bioremediation strategies in soils amended with sewage sludges.	[111]
112	2021	Kinetics and Stoichiometry of an Efficient Methanotroph Methylosarcina sp. LC-4 Isolated from a Municipal Solid Waste Dumpsite	[112]
113	2021	Assessment of heavy metal bioremediation potential of bacterial isolates from landfill soils.	[113]
114	2021	Landfill microbiome harbour plastic degrading genes: A metagenomic study of solid waste dumping site of Gujarat, India.	[114]
115	2021	Biodiversity of Actinomycetes from Heavy Metal Contaminated Technosols	[115]
116	2021	Influences of geochemical factors and substrate availability on Gram-positive and Gram-negative bacterial distribution and bio-processes in ageing municipal landfills.	[116]
117	2021	The Impacts of Different Biological Treatments on the Transformation of Explosives Waste Contaminated Sludge	[117]
118	2021	Diesel degradation efficiency of Enterobacter sp., Acinetobacter sp., and Cedecea sp. isolated from petroleum waste dumping site: a bioremediation view point.	[118]
119	2021	A review on disposal and utilization of phytoremediation plants containing heavy metals.	[119]
120	2022	Biodegradation of low density polyethylene (LDPE) by mesophilic fungus 'Penicillium citrinum' isolated from soils of plastic waste dump yard, Bhopal, India	[120]
121	2022	Ability of Urtica dioica L. to adsorb heavy metals (Pb, Cd, As, and Ni) from contaminated soils	[121]
122	2022	Insights into the effects of Fenton oxidation on PAH removal and indigenous bacteria in aged subsurface soil	[122]
123	2022	Diverse sustainable materials for the treatment of petroleum sludge and remediation of contaminated sites: a review	[123]
124	2022	Review on recent progress of bioremediation strategies in Landfill leachate - A green approach	[124]

125	2022	Bacterial strains found in the soils of a municipal solid waste dumping site facilitated phosphate solubilization along with cadmium remediation	[125]
126	2022	Biotechnological potential of microorganisms from landfill leachate: isolation, antibiotic resistance and leachate discoloration	[126]
127	2022	Insights on the bioremediation technologies for pesticide-contaminated soils	[127]
128	2022	Advances and Applications of Bioremediation: Network of Omics, System Biology, Gene Editing and Nanotechnology	[128]
129	2022	An Overview of Antibiotic Resistance and Abiotic Stresses Affecting Antimicrobial Resistance in Agricultural Soils	[129]
130	2022	Biochar Produced from Organic Waste Digestate and Its Potential Utilization for Soil Remediation: An Overview	[130]
131	2022	Phytoremediation of leachate contaminated soil: a biotechnical option for the bioreduction of heavy metals induced pollution in tropical landfill	[131]
132	2022	Preliminary investigation of microorganisms potentially involved in microplastics degradation using an integrated metagenomic and biochemical approach	[132]
133	2022	A laboratory-scale phytocover system for municipal solid waste landfills	[133]
134	2022	Converting wastes to resource: Utilization of dewatered municipal sludge for calcium-based biochar adsorbent preparation and land application as a fertilizer	[134]
135	2022	Vermitechnology: An Eco-Friendly Approach for Organic Solid Waste Management and Soil Fertility Improvement—A Review	[135]
136	2022	Exploring the plastic degrading ability of microbial communities through metagenomic approach	[136]
137	2022	Review on bioremediation and phytoremediation techniques of heavy metals in contaminated soil from dump site	[137]
138	2022	A comprehensive review on recent advancements in biodegradation and sustainable management of biopolymers	[138]
139	2022	Heavy metals removal from dumpsite leachate by algae and cyanobacteria	[139]
140	2022	Nitrogen addition facilitates phytoremediation of PAH-Cd cocontaminated dumpsite soil by altering alfalfa growth and rhizosphere communities	[140]
141	2022	Degradation of dibutyl phthalate by Paenarthrobacter sp. Shss isolated from Saravan landfill, Hyrcanian Forests, Iran.	[141]
142	2022	Bioprocess potential of Eco-friendly fungal isolates converting organic waste to bioresource.	[142]
143	2022	Contrasted microbial community colonization of a bauxite residue deposit marked by a complex geochemical context.	[143]

144	2022	Pyrolytic Remediation and Ecotoxicity Assessment of Fuel-Oil-Contaminated Soil	[144]
145	2022	Bioleaching of As from mine tailings using an autochthonous Bacillus cereus strain	[145]
146	2022	Membrane composition and successful bioaugmentation. Studies of the interactions of model thylakoid and plasma cyanobacterial and bacterial membranes with fungal membrane-lytic enzyme Lecitase ultra.	[146]
147	2022	Bioaugmentation-assisted bioremediation and kinetics modelling of heavy metal-polluted landfill soil	[147]
148	2022	A review on settlement models of municipal solid waste landfills.	[148]
149	2022	Micro-algae assisted green bioremediation of water pollutants rich leachate and source products recovery.	[149]
150	2022	A critical review on microbes-based treatment strategies for mitigation of toxic pollutants	[150]
151	2022	Coupling phytoremediation of Pb-contaminated soil and biomass energy production: A comparative Life Cycle Assessment.	[151]
152	2023	Interaction among Heavy Metals in Landfill Leachate and Their Effect on the Phytoremediation Process of Indian Marigold	[152]

References

[1] "Bioremediation of heavy metals from contaminated sites using potential species, a review".

[2] A. K. Awasthi, A. K. Pandey, and J. Khan, "A preliminary report of indigenous fungal isolates from contaminated municipal solid waste site in India," *Environmental Science and Pollution Research*, vol. 24, no. 9, pp. 8880–8888, Mar. 2017, doi: 10.1007/s11356-017-8472-0.

[3] O. B. Ojuederie and O. O. Babalola, "Microbial and plant-assisted bioremediation of heavy metal polluted environments: A review," *International Journal of Environmental Research and Public Health*, vol. 14, no. 12. MDPI, Dec. 04, 2017. doi: 10.3390/ijerph14121504.

[4] B. Jayanth, C. U. Emenike, P. Agamuthu, and S. H. Fauziah, "Potential of Cordyline sp Plant for Remediation of Metal-Leachate Contaminated Soil," *International Journal of Chemical Engineering and Applications*, vol. 8, no. 3, pp. 199–202, Jun. 2017, doi: 10.18178/ijcea.2017.8.3.656.

[5] C. U. Emenike, W. Liew, M. G. Fahmi, K. N. Jalil, A. Pariathamby, and F. S. Hamid, "Optimal Removal of Heavy Metals From Leachate Contaminated Soil Using Bioaugmentation Process," *Clean (Weinh)*, vol. 45, no. 2, Feb. 2017, doi: 10.1002/clen.201500802.

[6] S. Voběrková *et al.*, “Effect of inoculation with white-rot fungi and fungal consortium on the composting efficiency of municipal solid waste,” *Waste Management*, vol. 61, pp. 157–164, Mar. 2017, doi: 10.1016/j.wasman.2016.12.039.

[7] T. F. C. V. Silva *et al.*, “An innovative multistage treatment system for sanitary landfill leachate depuration: Studies at pilot-scale,” *Science of the Total Environment*, vol. 576, pp. 99–117, Jan. 2017, doi: 10.1016/j.scitotenv.2016.10.058.

[8] S. H. Fauziah, P. Agamuthu, R. Hashim, A. K. Izyani, and C. U. Emenike, “Assessing the bioaugmentation potentials of individual isolates from landfill on metal-polluted soil,” *Environ Earth Sci*, vol. 76, no. 11, Jun. 2017, doi: 10.1007/s12665-017-6739-x.

[9] B. Jayanthi, C. U. Emenike, S. H. Auta, P. Agamuthu, and S. H. Fauziah, “Characterization of induced metal responses of bacteria isolates from active non-sanitary landfill in Malaysia,” *Int Biodeterior Biodegradation*, vol. 119, pp. 467–475, Apr. 2017, doi: 10.1016/j.ibiod.2016.10.053.

[10] S. BOTEVA and Anelia. KENAROVA, “Impact of Methane Concentration and Temperature on the Activity of a Methanotrophic Strain Isolated from a Municipal Landfill.,” *Comptes rendus de l’Académie bulgare des Sciences*, vol. 70, no. 9, pp. 1271–1279, 2017.

[11] C. U. Emenike, P. Agamuthu, and S. H. Fauziah, “Sustainable remediation of heavy metal polluted soil: A biotechnical interaction with selected bacteria species,” *J Geochem Explor*, vol. 182, pp. 275–278, Nov. 2017, doi: 10.1016/j.gexplo.2016.10.002.

[12] S. Khalid *et al.*, “A comparison of technologies for remediation of heavy metal contaminated soils To cite this version A comparison of technologies for remediation of heavy metal contaminated soils,” *J Geochem Explor*, vol. 182, 2016, doi: 10.1016/j.gexplo.2016.11.021ï.

[13] H. M. Zhao *et al.*, “Enhanced dissipation of DEHP in soil and simultaneously reduced bioaccumulation of DEHP in vegetable using bioaugmentation with exogenous bacteria,” *Biol Fertil Soils*, vol. 53, no. 6, pp. 663–675, Aug. 2017, doi: 10.1007/s00374-017-1208-y.

[14] E. Morillo and J. Villaverde, “Advanced technologies for the remediation of pesticide-contaminated soils,” *Science of the Total Environment*, vol. 586. Elsevier B.V., pp. 576–597, May 15, 2017. doi: 10.1016/j.scitotenv.2017.02.020.

[15] R. González-Núñez, A. Rigol, and M. Vidal, “Assessing the efficacy over time of the addition of industrial by-products to remediate contaminated soils at a pilot-plant scale,” *Environ Monit Assess*, vol. 189, no. 4, p. 155, Apr. 2017, doi: 10.1007/s10661-017-5864-z.

[16] A. Dadrasnia, M. S. Azirun, and S. B. Ismail, “Optimal reduction of chemical oxygen demand and NH3-N from landfill leachate using a strongly resistant novel Bacillus salmalaya strain,” *BMC Biotechnol*, vol. 17, no. 1, Nov. 2017, doi: 10.1186/s12896-017-0395-9.

[17] A. Roxana Gabriela Popa, L. eng Emil Catalin Schiopu, L. Irina-Ramona Pecingina, and A. eng Cazalbasu Violeta Ramona, “Section Ecology and Environmental Protection APPLICATION OF BIOREMEDIATION IN THE DECONTAMINATION OF POLLUTED SOIL WITH PETROLEUM HYDROCARBONS”, doi: 10.5593/sgem2018/5.2.

[18] M. Kończak and P. Oleszczuk, “Application of biochar to sewage sludge reduces toxicity and improve organisms growth in sewage sludge-amended soil in long term field experiment,” *Science of the Total Environment*, vol. 625, pp. 8–15, Jun. 2018, doi: 10.1016/j.scitotenv.2017.12.118.

[19] M. Ohlbaum, S. L. Wadgaonkar, J. J. A. van Bruggen, Y. v. Nancharaiah, and P. N. L. Lens, “Phytoremediation of seleniferous soil leachate using the aquatic plants Lemna minor and Egeria densa,” *Ecol Eng*, vol. 120, pp. 321–328, Sep. 2018, doi: 10.1016/j.ecoleng.2018.06.013.

[20] N. Manoj Kumar and M. Chaithanya Sudha, “Characterization of Leachate and Groundwater in and Around Saduperi Municipal Solid Waste Open Dump Site, Vellore District, Tamil Nadu, India,” in *Energy, Environment, and Sustainability*, Springer Nature, 2018, pp. 279–299. doi: 10.1007/978-981-10-7413-4_15.

[21] Q. Liu *et al.*, “Bioremediation of petroleum-contaminated soil using aged refuse from landfills,” *Waste Management*, vol. 77, pp. 576–585, Jul. 2018, doi: 10.1016/j.wasman.2018.05.010.

[22] M. BŁASZAK and I. OCHMIAN, “THE NATIONAL PROGRAM FOR THE LIQUIDATION OF PESTICIDE WASTE LANDFILLS, SUCCESSES AND UNUSED OPPORTUNITIES – CASE STUDY FROM POLAND,” *Folia Pomeranae Universitatis Technologiae Stetinensis Agricultura, Alimentaria, Piscaria et Zootechnica*, vol. 345, no. 48, pp. 15–26, Dec. 2018, doi: 10.21005/AAPZ2018.48.4.02.

[23] C. U. Emenike, B. Jayanthi, P. Agamuthu, and S. H. Fauziah, “BIOTRANSFORMATION AND REMOVAL OF HEAVY METALS: A REVIEW OF PHYTOREMEDIATION AND MICROBIAL REMEDIATION ASSESSMENT ON CONTAMINATED SOIL.” [Online]. Available: https://mc06.manuscriptcentral.com/er-pubs

[24] J. S. Adriano, G. G. Oyong, E. C. Cabrera, and J. I. B. Janairo, “Screening of Silver-Tolerant Bacteria from a Major Philippine Landfill as Potential Bioremediation Agents,” *Ecological Chemistry and Engineering S*, vol. 25, no. 3, pp. 469–485, Sep. 2018, doi: 10.1515/eces-2018-0032.

[25] A. , A. M. S. , M. S. , A. I. , K. A. , H. R. Jamil, “Raoultella and Enterococcus species identified as high chromium and arsenic tolerant bacteria,” *Biointerface Res Appl Chem*, vol. 8, no. 3, 2018.

[26] A. Paskuliakova, T. McGowan, S. Tonry, and N. Touzet, “Phycoremediation of landfill leachate with the chlorophyte Chlamydomonas sp. SW15aRL and evaluation of toxicity pre and post treatment,” *Ecotoxicol Environ Saf*, vol. 147, pp. 622–630, Jan. 2018, doi: 10.1016/j.ecoenv.2017.09.010.

[27] M. Y. Zainun and K. Simarani, “Metagenomics profiling for assessing microbial diversity in both active and closed landfills,” *Science of the Total Environment*, vol. 616–617, pp. 269–278, Mar. 2018, doi: 10.1016/j.scitotenv.2017.10.266.

[28] S. Collado, P. Oulego, O. Suárez-Iglesias, and M. Díaz, “Biodegradation of dissolved humic substances by fungi,” *Applied Microbiology and Biotechnology*, vol. 102, no. 8. Springer Verlag, pp. 3497–3511, Apr. 01, 2018. doi: 10.1007/s00253-018-8851-6.

[29] S. Dey and P. Tribedi, “Microbial functional diversity plays an important role in the degradation of polyhydroxybutyrate (PHB) in soil,” *3 Biotech*, vol. 8, no. 3, Mar. 2018, doi: 10.1007/s13205-018-1201-7.

[30] I. V. K. Nayanthika, D. T. Jayawardana, N. J. G. J. Bandara, P. M. Manage, and R. M. T. D. Madushanka, “Effective use of iron-aluminum rich laterite based soil mixture for treatment of landfill leachate,” *Waste Management*, vol. 74, pp. 347–361, Apr. 2018, doi: 10.1016/j.wasman.2018.01.013.

[31] W. Guidi Nissim *et al.*, “Phytoremediation of sewage sludge contaminated by trace elements and organic compounds,” *Environ Res*, vol. 164, pp. 356–366, Jul. 2018, doi: 10.1016/J.ENVRES.2018.03.009.

[32] A. Rajasekar, R. Sekar, E. Medina-Roldan, J. Bridge, C. K. S. Moy, and S. Wilkinson, “Next-generation sequencing showing potential leachate influence on bacterial communities around a landfill in China.” [Online]. Available: www.nrcresearchpress.com

[33] V. Sharma and D. Pant, “Biocompatible metal decontamination from soil using Ageratum conyzoides,” *Environmental Science and Pollution Research*, vol. 25, no. 22, pp. 22294–22307, Aug. 2018, doi: 10.1007/s11356-018-2343-1.

[34] N. Bhatnagar, “ ROLE OF MICROBES IN BIOREMEDIATION OF POLLUTANTS,” *Microbes in Agriculture and Environmental Development*, vol. 0, no. 0, 2018.

[35] J. Rajasekar, E. Medina-Roldan, C. K. S. Moy, and J. , Rajasekar, “Biomineralisation performance of bacteria isolated from a landfill in China.” [Online]. Available: http://shura.shu.ac.uk/22438/

[36] J. Ye, X. Chen, C. Chen, and B. Bate, “Emerging sustainable technologies for remediation of soils and groundwater in a municipal solid waste landfill site – A review,” *Chemosphere*, vol. 227, pp. 681–702, Jul. 2019, doi: 10.1016/j.chemosphere.2019.04.053.

[37] E. Coppini *et al.*, “Design and start-up of a constructed wetland as tertiary treatment for landfill leachates,” *Water Science and Technology*, vol. 79, no. 1, pp. 145–155, Jan. 2019, doi: 10.2166/wst.2019.030.

[38] W. Hu *et al.*, “Enhanced degradation of 1-naphthol in landfill leachate using Arthrobacter sp.,” *Environmental Technology (United Kingdom)*, vol. 40, no. 7, pp. 835–842, Mar. 2019, doi: 10.1080/09593330.2017.1408695.

[39] O. Oziegbe, O. T. Aladesanmi, and O. O. Awotoye, “Effect of biochar on the nutrient contents and metal recovery efficiency in sorghum planted on landfill soils,” *International Journal of Environmental Science and Technology*, vol. 16, no. 5, pp. 2259–2270, May 2019, doi: 10.1007/s13762-018-1843-3.

[40] R. Bian, W. Shi, Y. Duan, and X. Chai, "Effect of soil types and ammonia concentrations on the contribution of ammonia-oxidizing bacteria to CH4 oxidation," *Waste Management and Research*, vol. 37, no. 7, pp. 698–705, Jul. 2019, doi: 10.1177/0734242X19843988.

[41] S. Collado, P. Oulego, O. Suárez-Iglesias, and M. Díaz, "Leachates and natural organic matter. A review of their biotreatment using fungi," *Waste Management*, vol. 96. Elsevier Ltd, pp. 108–120, Aug. 01, 2019. doi: 10.1016/j.wasman.2019.07.018.

[42] L. Silvani *et al.*, "Can biochar and designer biochar be used to remediate per- and polyfluorinated alkyl substances (PFAS) and lead and antimony contaminated soils?," *Science of the Total Environment*, vol. 694, Dec. 2019, doi: 10.1016/j.scitotenv.2019.133693.

[43] F. Spina *et al.*, "OPTIMIZING BIOREMEDIATION OF HYDROCARBONPOLLUTED SOIL BY LIFE CYCLE ASSESSMENT (LCA) APPROACH," 2018. [Online]. Available: http://www.eemj.icpm.tuiasi.ro/;http://www.eemj.eu

[44] E. Koshlaf, E. Shahsavari, N. Haleyur, A. Mark Osborn, and A. S. Ball, "Effect of biostimulation on the distribution and composition of the microbial community of a polycyclic aromatic hydrocarbon-contaminated landfill soil during bioremediation," *Geoderma*, vol. 338, pp. 216–225, Mar. 2019, doi: 10.1016/j.geoderma.2018.12.001.

[45] E. Puglisi, F. Romaniello, S. Galletti, E. Boccaleri, A. Frache, and P. S. Cocconcelli, "Selective bacterial colonization processes on polyethylene waste samples in an abandoned landfill site," *Sci Rep*, vol. 9, no. 1, Dec. 2019, doi: 10.1038/s41598-019-50740-w.

[46] J. Sedlakova-Kadukova, A. Kopcakova, L. Gresakova, A. Godany, and P. Pristas, "Bioaccumulation and biosorption of zinc by a novel Streptomyces K11 strain isolated from highly alkaline aluminium brown mud disposal site," *Ecotoxicol Environ Saf*, vol. 167, pp. 204–211, Jan. 2019, doi: 10.1016/j.ecoenv.2018.09.123.

[47] M. Taran, R. Fateh, S. Rezaei, and M. K. Gholi, "Isolation of arsenic accumulating bacteria from garbage leachates for possible application in bioremediation," 2019. [Online]. Available: http://ijm.tums.ac.ir

[48] S. jia Liu *et al.*, "Succession and diversity of microbial communities in landfills with depths and ages and its association with dissolved organic matter and heavy metals," *Science of the Total Environment*, vol. 651, pp. 909–916, Feb. 2019, doi: 10.1016/j.scitotenv.2018.09.267.

[49] E. Fasani, G. DalCorso, A. Zerminiani, A. Ferrarese, P. Campostrini, and A. Furini, "Phytoremediatory efficiency of Chrysopogon zizanioides in the treatment of landfill leachate: a case study," *Environmental Science and Pollution Research*, vol. 26, no. 10, pp. 10057–10069, Apr. 2019, doi: 10.1007/s11356-019-04505-7.

[50] M. Vargas-Suárez, V. Fernández-Cruz, and H. Loza-Tavera, "Biodegradation of polyacrylic and polyester polyurethane coatings by enriched microbial communities," *Appl Microbiol Biotechnol*, vol. 103, no. 7, pp. 3225–3236, Apr. 2019, doi: 10.1007/s00253-019-09660-y.

[51] P. Shrestha, K. Bellitürk, and J. Görres, "Phytoremediation of Heavy Metal-Contaminated Soil by Switchgrass: A Comparative Study Utilizing Different Composts and Coir Fiber on

Pollution Remediation, Plant Productivity, and Nutrient Leaching," *Int J Environ Res Public Health*, vol. 16, no. 7, p. 1261, Apr. 2019, doi: 10.3390/ijerph16071261.

[52] Y. Tao *et al.*, "Metabolism of diethyl phthalate (DEP) and identification of degradation intermediates by Pseudomonas sp. DNE-S1," *Ecotoxicol Environ Saf*, vol. 173, pp. 411–418, May 2019, doi: 10.1016/j.ecoenv.2019.02.055.

[53] D. K. R. Bardají, J. P. R. Furlan, and E. G. Stehling, "Isolation of a polyethylene degrading Paenibacillus sp. from a landfill in Brazil," *Arch Microbiol*, vol. 201, no. 5, pp. 699–704, Jul. 2019, doi: 10.1007/s00203-019-01637-9.

[54] C. Lu *et al.*, "A PAH-degrading bacterial community enriched with contaminated agricultural soil and its utility for microbial bioremediation," *Environmental Pollution*, vol. 251, pp. 773–782, Aug. 2019, doi: 10.1016/j.envpol.2019.05.044.

[55] D. O'Connor *et al.*, "Phytoremediation: Climate change resilience and sustainability assessment at a coastal brownfield redevelopment," *Environ Int*, vol. 130, Sep. 2019, doi: 10.1016/j.envint.2019.104945.

[56] A. Hassan, A. Pariatamby, A. Ahmed, H. S. Auta, and F. S. Hamid, "Enhanced Bioremediation of Heavy Metal Contaminated Landfill Soil Using Filamentous Fungi Consortia: a Demonstration of Bioaugmentation Potential," *Water Air Soil Pollut*, vol. 230, no. 9, Sep. 2019, doi: 10.1007/s11270-019-4227-5.

[57] D. Daniel, P. Jegathambal, and B. Bevers, "In Situ Bioremediation of Textile Dye Effluent-Contaminated Soils Using Mixed Microbial Culture," *International Journal of Civil Engineering*, vol. 17, no. 10, pp. 1527–1536, Oct. 2019, doi: 10.1007/s40999-019-00414-5.

[58] M. A. Jóźwiak, M. Jóźwiak, R. Kozłowski, and M. Żelezik, "Zooremediation of leachates from municipal waste using Eisenia fetida (SAV.)," *Environmental Pollution*, vol. 254, Nov. 2019, doi: 10.1016/j.envpol.2019.07.039.

[59] N. Sepehrnia *et al.*, "Transport, retention, and release of Escherichia coli and Rhodococcus erythropolis through dry natural soils as affected by water repellency," *Science of the Total Environment*, vol. 694, Dec. 2019, doi: 10.1016/j.scitotenv.2019.133666.

[60] Swati, P. Ghosh, and I. S. Thakur, "Biodegradation of pyrene by Pseudomonas sp. ISTPY2 isolated from landfill soil: Process optimisation using Box-Behnken design model," *Bioresour Technol Rep*, vol. 8, p. 100329, Dec. 2019, doi: 10.1016/j.biteb.2019.100329.

[61] F. Elbehiry, H. Elbasiouny, R. Ali, and E. C. Brevik, "Enhanced Immobilization and Phytoremediation of Heavy Metals in Landfill Contaminated Soils," *Water Air Soil Pollut*, vol. 231, no. 5, May 2020, doi: 10.1007/s11270-020-04493-2.

[62] A. Herrera-Quiterio *et al.*, "Antagonic and plant growth-promoting effects of bacteria isolated from mine tailings at El Fraile, Mexico," *Rev Argent Microbiol*, vol. 52, no. 3, pp. 231–239, Jul. 2020, doi: 10.1016/j.ram.2019.08.003.

[63] D. R. Meyer-Dombard, J. E. Bogner, and J. Malas, “A Review of Landfill Microbiology and Ecology: A Call for Modernization With ‘Next Generation’ Technology,” *Frontiers in Microbiology*, vol. 11. Frontiers Media S.A., Jun. 03, 2020. doi: 10.3389/fmicb.2020.01127.

[64] J. Ru, Y. Huo, and Y. Yang, “Microbial Degradation and Valorization of Plastic Wastes,” *Frontiers in Microbiology*, vol. 11. Frontiers Media S.A., Apr. 21, 2020. doi: 10.3389/fmicb.2020.00442.

[65] L. Qin, Z. Xu, L. Liu, H. Lu, Y. Wan, and Q. Xue, “In-situ biodegradation of volatile organic compounds in landfill by sewage sludge modified waste-char,” *Waste Management*, vol. 105, pp. 317–327, Mar. 2020, doi: 10.1016/j.wasman.2020.02.022.

[66] B. Arab, F. Hassanpour, M. Arshadi, S. Yaghmaei, and J. Hamedi, “Optimized bioleaching of copper by indigenous cyanogenic bacteria isolated from the landfill of e-waste,” *J Environ Manage*, vol. 261, May 2020, doi: 10.1016/j.jenvman.2020.110124.

[67] M. E. Karim *et al.*, “Biotransformation of chromium (VI) by Bacillus sp. isolated from chromate contaminated landfill site,” *Chemistry and Ecology*, vol. 36, no. 10, pp. 922–937, Nov. 2020, doi: 10.1080/02757540.2020.1799992.

[68] N. Salehi, A. Azhdarpoor, and M. Shirdarreh, “The effect of different levels of leachate on phytoremediation of pyrene-contaminated soil and simultaneous extraction of lead and cadmium,” *Chemosphere*, vol. 246, May 2020, doi: 10.1016/j.chemosphere.2020.125845.

[69] U. Song, “Improvement of soil properties and plant responses by compost generated from biomass of phytoremediation plant,” *Environmental Engineering Research*, vol. 25, no. 5, pp. 638–644, Oct. 2020, doi: 10.4491/eer.2019.59.

[70] C. O. Asadu *et al.*, “Investigation of the influence of biofertilizer synthesized using microbial inoculums on the growth performance of two agricultural crops,” *Biotechnology Reports*, vol. 27, Sep. 2020, doi: 10.1016/j.btre.2020.e00493.

[71] T. Steliga, K. Wojtowicz, P. Kapusta, and J. Brzeszcz, “Assessment of biodegradation efficiency of polychlorinated biphenyls (PCBs) and petroleum hydrocarbons (TPH) in soil using three individual bacterial strains and their mixed culture,” *Molecules*, vol. 25, no. 3, Feb. 2020, doi: 10.3390/molecules25030709.

[72] Swati, M. Kumari, P. Ghosh, and I. S. Thakur, “Evaluation of a biosurfactant producing bacterial strain Pseudomonas sp. ISTPY2 for efficient pyrene degradation and landfill soil bioremediation through soil microcosm and proteomic studies,” *Bioresour Technol Rep*, vol. 12, Dec. 2020, doi: 10.1016/j.biteb.2020.100607.

[73] I. G. S. da Silva, F. C. G. de Almeida, N. M. P. da Rocha e Silva, A. A. Casazza, A. Converti, and L. A. Sarubbo, “Soil bioremediation: Overview of technologies and trends,” *Energies*, vol. 13, no. 18. MDPI AG, Sep. 01, 2020. doi: 10.3390/en13184664.

[74] Y. Zegzouti *et al.*, “Bioremediation of landfill leachate by Aspergillus flavus in submerged culture: Evaluation of the process efficiency by physicochemical methods and 3D

fluorescence spectroscopy," *J Environ Manage*, vol. 255, Feb. 2020, doi: 10.1016/j.jenvman.2019.109821.

[75] A. Hassan, A. Periathamby, A. Ahmed, O. Innocent, and F. S. Hamid, "Effective bioremediation of heavy metal–contaminated landfill soil through bioaugmentation using consortia of fungi," *J Soils Sediments*, vol. 20, no. 1, pp. 66–80, Jan. 2020, doi: 10.1007/s11368-019-02394-4.

[76] D. Li *et al.*, "Discovery of a novel native bacterium of Providencia sp. with high biosorption and oxidation ability of manganese for bioleaching of heavy metal contaminated soils," *Chemosphere*, vol. 241, Feb. 2020, doi: 10.1016/j.chemosphere.2019.125039.

[77] K. Thakur *et al.*, "Bioprospecting potential of microbial communities in solid waste landfills for novel enzymes through metagenomic approach," *World J Microbiol Biotechnol*, vol. 36, no. 3, Mar. 2020, doi: 10.1007/s11274-020-02812-7.

[78] L. Qin, X. Huang, Q. Xue, L. Liu, and Y. Wan, "In-situ biodegradation of harmful pollutants in landfill by sludge modified biochar used as biocover," *Environmental Pollution*, vol. 258, Mar. 2020, doi: 10.1016/j.envpol.2019.113710.

[79] P. Chakraborty, R. Dave, P. Paul, S. Dutta, S. Sarkar, and P. Tribedi, "Exploration of strategies to increase the nitrogen and phosphate content of solid waste landfill soil," *Environ Monit Assess*, vol. 192, no. 4, Apr. 2020, doi: 10.1007/s10661-020-8200-y.

[80] Q. Xu, S. Renault, D. Goltz, and Q. Yuan, "Phytoremediation of waste dumping site soil and landfill leachate by using cattail (Typha latifolia)," *Environmental Technology (United Kingdom)*, vol. 41, no. 9, pp. 1101–1106, Apr. 2020, doi: 10.1080/09593330.2018.1521474.

[81] O. Aromolaran, O. E. Fagade, H. M. Olarenwaju, and A. A. Ogunjobi, "Degradation of di-(2-ethylhexyl) phthalate by Bacillus aquimaris isolated from Ajakanga municipal solid waste leachate," *Malays J Microbiol*, vol. 16, no. 2, pp. 117–123, 2020, doi: 10.21161/mjm.190433.

[82] A. Hassan, A. Pariatamby, I. C. Ossai, and F. S. Hamid, "Bioaugmentation assisted mycoremediation of heavy metal and/metalloid landfill contaminated soil using consortia of filamentous fungi," *Biochem Eng J*, vol. 157, Apr. 2020, doi: 10.1016/j.bej.2020.107550.

[83] Y. Zegzouti *et al.*, "Screening and selection of autochthonous fungi from leachate contaminated-soil for bioremediation of different types of leachate," *Environmental Engineering Research*, vol. 25, no. 5, pp. 722–734, 2020, doi: 10.4491/eer.2019.317.

[84] K. Janczak, G. B. Dąbrowska, A. Raszkowska-Kaczor, D. Kaczor, K. Hrynkiewicz, and A. Richert, "Biodegradation of the plastics PLA and PET in cultivated soil with the participation of microorganisms and plants," *Int Biodeterior Biodegradation*, vol. 155, Nov. 2020, doi: 10.1016/j.ibiod.2020.105087.

[85] S. J. Liu, M. X. Zheng, X. J. Sun, B. D. Xi, X. S. He, and X. Xiao, "Evolution properties and dechlorination capacities of particulate organic matter from a landfill," *J Hazard Mater*, vol. 400, Dec. 2020, doi: 10.1016/j.jhazmat.2020.123313.

[86] F. C. Mihai, R. Plana, M. J. Taherzadeh, M. K. Aswathi, and C. Ezeah, “Bioremediation of organic contaminants based on biowaste composting practices,” in *Handbook of Bioremediation: Physiological, Molecular and Biotechnological Interventions*, Elsevier, 2020, pp. 701–714. doi: 10.1016/B978-0-12-819382-2.00045-4.

[87] H. Raclavská *et al.*, “EFFECT OF BIOCHAR ADDITION ON THE IMPROVEMENT OF THE QUALITY PARAMETERS OF COMPOST USED FOR LAND RECLAMATION.”

[88] A. Mojiri *et al.*, “Treatment of landfill leachate with different techniques: An overview,” *Journal of Water Reuse and Desalination*, vol. 11, no. 1, pp. 66–96, 2021, doi: 10.2166/wrd.2020.079.

[89] O. B. Ojuederie and O. O. Babalola, “Microbial and plant-assisted bioremediation of heavy metal polluted environments: A review,” *International Journal of Environmental Research and Public Health*, vol. 14, no. 12. MDPI, Dec. 04, 2017. doi: 10.3390/ijerph14121504.

[90] Z. Soleimani, S. Gharavi, M. Soudi, and Z. Moosavi-Nejad, “A survey of intact low-density polyethylene film biodegradation by terrestrial Actinobacterial species,” *International Microbiology*, vol. 24, no. 1, pp. 65–73, Jan. 2021, doi: 10.1007/s10123-020-00142-0.

[91] M. Mewada, S. Albert, A. Taunk, and K. Bhatt, “Screening of Fungal Microbiome to Identify Potential Polyethylene Degrading Fungi,” *The Journal of Solid Waste Technology and Management*, vol. 47, no. 4, pp. 619–626, Nov. 2021, doi: 10.5276/JSWTM/2021.619.

[92] M. Ajona and P. Vasanthi, “Bioremediation of petroleum contaminated soils – A review,” in *Materials Today: Proceedings*, 2020, vol. 45, pp. 7117–7122. doi: 10.1016/j.matpr.2021.01.949.

[93] R. J. Cabello-Torres, J. R. Romero-Longwell, L. Valdiviezo-Gonzales, R. Munive-Cerrón, and C. A. Castañeda-Olivera, “Biochar derived from pig manure with ability to reduce the availability of Pb in contaminated agricultural soils,” *Scientia Agropecuaria*, vol. 12, no. 4, pp. 461–470, Oct. 2021, doi: 10.17268/sci.agropecu.2021.050.

[94] I. A. Allamin and M. Y. Shukor, “Phytoremediation of PAHs in Contaminated Soils: A Review,” *Bioremediation Science and Technology Research*, vol. 9, no. 2, pp. 1–6, Dec. 2021, doi: 10.54987/bstr.v9i2.609.

[95] K. Mazumdar and S. Das, “Phytoremediation of soil treated with metalliferous leachate from an abandoned industrial site by Alternanthera sessilis and Ipomoea aquatica: Metal extraction and biochemical responses,” *Ecol Eng*, vol. 170, p. 106349, Nov. 2021, doi: 10.1016/j.ecoleng.2021.106349.

[96] “Environmental risk assessment in selected dumpsites in Abakaliki metropolis, Ebonyi state, southeastern Nigeria”.

[97] N. Kennedy, R. D. Lally, S. W. Walsh, D. N. Dowling, and D. Ryan, “Effect of green waste and lime amendments on biostabilisation, physical-chemical and microbial properties of the composted fine fraction of residual municipal solid waste,” *Waste Management and Research*, vol. 39, no. 8, pp. 1069–1077, Aug. 2021, doi: 10.1177/0734242X21996823.

[98] D. Padhan, P. P. Rout, R. Kundu, S. Adhikary, and P. P. Padhi, "Bioremediation of Heavy Metals and Other Toxic Substances by Microorganisms," in *Soil Bioremediation*, Wiley, 2021, pp. 285–329. doi: 10.1002/9781119547976.ch12.

[99] H. T. Tran *et al.*, "Aerobic composting remediation of petroleum hydrocarbon-contaminated soil. Current and future perspectives," *Science of the Total Environment*, vol. 753, Jan. 2021, doi: 10.1016/j.scitotenv.2020.142250.

[100] U. Anand *et al.*, "Potential environmental and human health risks caused by antibiotic-resistant bacteria (ARB), antibiotic resistance genes (ARGs) and emerging contaminants (ECs) from municipal solid waste (MSW) landfill," *Antibiotics*, vol. 10, no. 4, Apr. 2021, doi: 10.3390/antibiotics10040374.

[101] F. Spina *et al.*, "Low density polyethylene degradation by filamentous fungi," *Environmental Pollution*, vol. 274, Apr. 2021, doi: 10.1016/j.envpol.2021.116548.

[102] C. I. Mawang, A. S. Azman, A. S. M. Fuad, and M. Ahamad, "Actinobacteria: An eco-friendly and promising technology for the bioaugmentation of contaminants," *Biotechnology Reports*, vol. 32. Elsevier B.V., Dec. 01, 2021. doi: 10.1016/j.btre.2021.e00679.

[103] M. Chakrabartty and G. Mohammad Harun-Or-Rashid, "Feasibility Study of the Soil Remediation Technologies in the Natural Environment," *American Journal of Civil Engineering*, vol. 9, no. 4, p. 91, 2021, doi: 10.11648/j.ajce.20210904.11.

[104] X. Li, J. Li, C. Qu, T. Yu, and M. Du, "Bioremediation of clay with high oil content and biological response after restoration," *Sci Rep*, vol. 11, no. 1, Dec. 2021, doi: 10.1038/s41598-021-88033-w.

[105] S. Paliya, A. Mandpe, M. S. Kumar, and S. Kumar, "Aerobic degradation of decabrominated diphenyl ether through a novel bacterium isolated from municipal waste dumping site: Identification, degradation and metabolic pathway," *Bioresour Technol*, vol. 333, Aug. 2021, doi: 10.1016/j.biortech.2021.125208.

[106] P. Wang, J. Gao, Y. Zhao, M. Zhang, and S. Zhou, "Biodegradability of di-(2-ethylhexyl) phthalate by a newly isolated bacterium Achromobacter sp. RX," *Science of the Total Environment*, vol. 755, Feb. 2021, doi: 10.1016/j.scitotenv.2020.142476.

[107] S. Khan, M. Naushad, E. C. Lima, S. Zhang, S. M. Shaheen, and J. Rinklebe, "Global soil pollution by toxic elements: Current status and future perspectives on the risk assessment and remediation strategies – A review," *J Hazard Mater*, vol. 417, Sep. 2021, doi: 10.1016/j.jhazmat.2021.126039.

[108] W. Guidi Nissim, E. Palm, C. Pandolfi, S. Mancuso, and E. Azzarello, "Relationship between Leachate Pollution Index and growth response of two willow and poplar hybrids: Implications for phyto-treatment applications," *Waste Management*, vol. 136, pp. 162–173, Dec. 2021, doi: 10.1016/j.wasman.2021.09.012.

[109] M. L. Huang, P. L. Yen, and V. H. C. Liao, "A combined approach to remediate cadmium contaminated sediment using the acidophilic sulfur-oxidizing bacterial SV5 and untreated

coffee ground,” *Chemosphere*, vol. 273, Jun. 2021, doi: 10.1016/j.chemosphere.2021.129662.

[110] M. F. Imron, S. B. Kurniawan, and S. R. S. Abdullah, “Resistance of bacteria isolated from leachate to heavy metals and the removal of Hg by Pseudomonas aeruginosa strain FZ-2 at different salinity levels in a batch biosorption system,” *Sustainable Environment Research*, vol. 31, no. 1, Dec. 2021, doi: 10.1186/s42834-021-00088-6.

[111] E. Urionabarrenetxea *et al.*, “Application of in situ bioremediation strategies in soils amended with sewage sludges,” *Science of the Total Environment*, vol. 766, Apr. 2021, doi: 10.1016/j.scitotenv.2020.144099.

[112] S. Nivedita, D. N. Arnepalli, and C. Krishnan, “Kinetics and Stoichiometry of an Efficient Methanotroph *Methylosarcina* sp. LC-4 Isolated from a Municipal Solid Waste Dumpsite,” *Journal of Environmental Engineering*, vol. 147, no. 5, May 2021, doi: 10.1061/(ASCE)EE.1943-7870.0001867.

[113] O. Oziegbe, A. O. Oluduro, E. J. Oziegbe, E. F. Ahuekwe, and S. J. Olorunsola, “Assessment of heavy metal bioremediation potential of bacterial isolates from landfill soils,” *Saudi J Biol Sci*, vol. 28, no. 7, pp. 3948–3956, Jul. 2021, doi: 10.1016/j.sjbs.2021.03.072.

[114] R. Kumar *et al.*, “Landfill microbiome harbour plastic degrading genes: A metagenomic study of solid waste dumping site of Gujarat, India,” *Science of the Total Environment*, vol. 779, Jul. 2021, doi: 10.1016/j.scitotenv.2021.146184.

[115] M. Cimermanova, P. Pristas, and M. Piknova, “Biodiversity of actinomycetes from heavy metal contaminated technosols,” *Microorganisms*, vol. 9, no. 8, Aug. 2021, doi: 10.3390/microorganisms9081635.

[116] L. Sekhohola-Dlamini, P. Dlamini, R. Selvarajan, H. J. O. Ogola, and M. Tekere, “Influences of geochemical factors and substrate availability on Gram-positive and Gram-negative bacterial distribution and bio-processes in ageing municipal landfills,” *International Microbiology*, vol. 24, no. 3, pp. 311–324, Aug. 2021, doi: 10.1007/s10123-021-00167-z.

[117] A. Aburto-medina, E. Shahsavari, M. Taha, A. Bates, L. van Ieperen, and A. S. Ball, “The impacts of different biological treatments on the transformation of explosives waste contaminated sludge,” *Molecules*, vol. 26, no. 16, Aug. 2021, doi: 10.3390/molecules26164814.

[118] I. Jerin *et al.*, “Diesel degradation efficiency of Enterobacter sp., Acinetobacter sp., and Cedecea sp. isolated from petroleum waste dumping site: a bioremediation view point,” *Arch Microbiol*, vol. 203, no. 8, pp. 5075–5084, Oct. 2021, doi: 10.1007/s00203-021-02469-2.

[119] Z. Liu and K. Q. Tran, “A review on disposal and utilization of phytoremediation plants containing heavy metals,” *Ecotoxicology and Environmental Safety*, vol. 226. Academic Press, Dec. 15, 2021. doi: 10.1016/j.ecoenv.2021.112821.

[120] S. Khan, S. A. Ali, and A. S. Ali, "Biodegradation of low density polyethylene (LDPE) by mesophilic fungus ' *Penicillium citrinum* ' isolated from soils of plastic waste dump yard, Bhopal, India," *Environ Technol*, pp. 1–15, Feb. 2022, doi: 10.1080/09593330.2022.2027025.

[121] K. Sharifi, A. Rahnavard, K. Saeb, F. Gholamreza Fahimi, and A. Tavana, "Ability of *Urtica dioica* L. to adsorb heavy metals (Pb, Cd, As, and Ni) from contaminated soils," *Soil and Sediment Contamination: An International Journal*, vol. 32, no. 1, pp. 51–84, Jan. 2023, doi: 10.1080/15320383.2022.2052263.

[122] Y. Gou, J. Ma, S. Yang, and Y. Song, "Insights into the effects of Fenton oxidation on PAH removal and indigenous bacteria in aged subsurface soil," *Environmental Pollution*, vol. 298, p. 118872, Apr. 2022, doi: 10.1016/j.envpol.2022.118872.

[123] "Diverse sustainable materials for the treatment of petroleum sludge and remediation of contaminated sites a review".

[124] B. Tan *et al.*, "Review on recent progress of bioremediation strategies in Landfill leachate - A green approach," *Journal of Water Process Engineering*, vol. 50. Elsevier Ltd, Dec. 01, 2022. doi: 10.1016/j.jwpe.2022.103229.

[125] S. Sahu *et al.*, "Bacterial strains found in the soils of a municipal solid waste dumping site facilitated phosphate solubilization along with cadmium remediation," *Chemosphere*, vol. 287, p. 132320, Jan. 2022, doi: 10.1016/J.CHEMOSPHERE.2021.132320.

[126] L. A. A. Garcete, J. E. R. Martinez, D. B. V. Barrera, R. C. Bonugli-Santos, and M. R. Z. Passarini, "Biotechnological potential of microorganisms from landfill leachate: isolation, antibiotic resistance and leachate discoloration," *An Acad Bras Cienc*, vol. 94, no. 3, 2022, doi: 10.1590/0001-3765202220210642.

[127] H. , M. S. , A. L. B. , M. E. , R. V. D. , M. T. , & A. T. Karimi, "Insights on the bioremediation technologies for pesticide-contaminated soils.," *Environ Geochem Health*, vol. 44, no. 4, pp. 1329–1354, 2022.

[128] R. Chakraborty, S. Karmakar, and W. Ansar, "Advances and Applications of Bioremediation: Network of Omics, System Biology, Gene Editing and Nanotechnology," in *Environmental Informatics*, Singapore: Springer Nature Singapore, 2022, pp. 167–199. doi: 10.1007/978-981-19-2083-7_10.

[129] A. K. Rad *et al.*, "An Overview of Antibiotic Resistance and Abiotic Stresses Affecting Antimicrobial Resistance in Agricultural Soils," *International Journal of Environmental Research and Public Health*, vol. 19, no. 8. MDPI, Apr. 01, 2022. doi: 10.3390/ijerph19084666.

[130] S. Wongrod *et al.*, "Biochar Produced from Organic Waste Digestate and Its Potential Utilization for Soil Remediation: An Overview," 2022, pp. 263–292. doi: 10.1007/978-3-030-87633-3_10.

[131] J. Barasarathi, H. Auwal, A. Pariatamby, F. S. Hamid, and E. C. Uche, "Phytoremediation of leachate contaminated soil: a biotechnical option for the bioreduction of heavy metals

induced pollution in tropical landfill," *Environmental Science and Pollution Research*, vol. 29, no. 15, pp. 22069–22081, Mar. 2022, doi: 10.1007/s11356-021-17389-3.

[132] G. Giangeri *et al.*, "Preliminary investigation of microorganisms potentially involved in microplastics degradation using an integrated metagenomic and biochemical approach," *Science of The Total Environment*, vol. 843, p. 157017, Oct. 2022, doi: 10.1016/j.scitotenv.2022.157017.

[133] A. Khapre, S. A. Khan, and S. Kumar, "A laboratory-scale phytocover system for municipal solid waste landfills," *Environmental Technology (United Kingdom)*, vol. 43, no. 24, pp. 3670–3681, 2022, doi: 10.1080/09593330.2021.1931470.

[134] J. Qin, C. Zhang, Z. Chen, X. Wang, Y. Zhang, and L. Guo, "Converting wastes to resource: Utilization of dewatered municipal sludge for calcium-based biochar adsorbent preparation and land application as a fertilizer," *Chemosphere*, vol. 298, p. 134302, Jul. 2022, doi: 10.1016/j.chemosphere.2022.134302.

[135] M. A. Siddiqui, A. Neeraj, and R. Y. Hiranmai, "Vermitechnology: An Eco-Friendly Approach for Organic Solid Waste Management and Soil Fertility Improvement—A Review," in *Strategies and Tools for Pollutant Mitigation*, Cham: Springer International Publishing, 2022, pp. 91–112. doi: 10.1007/978-3-030-98241-6_5.

[136] Shilpa, N. Basak, and S. S. Meena, "Exploring the plastic degrading ability of microbial communities through metagenomic approach," *Mater Today Proc*, vol. 57, pp. 1924–1932, 2022, doi: 10.1016/j.matpr.2022.02.308.

[137] R. Praveen and R. Nagalakshmi, "Review on bioremediation and phytoremediation techniques of heavy metals in contaminated soil from dump site," *Mater Today Proc*, Jan. 2022, doi: 10.1016/j.matpr.2022.07.190.

[138] S. K. Awasthi *et al.*, "A comprehensive review on recent advancements in biodegradation and sustainable management of biopolymers," *Environmental Pollution*, vol. 307, p. 119600, Aug. 2022, doi: 10.1016/j.envpol.2022.119600.

[139] J. Iqbal, A. Javed, and M. A. Baig, "Heavy metals removal from dumpsite leachate by algae and cyanobacteria," *Bioremediation Journal*, vol. 26, no. 1. Taylor and Francis Ltd., pp. 31–40, 2022. doi: 10.1080/10889868.2021.1884530.

[140] Y. Li *et al.*, "Nitrogen addition facilitates phytoremediation of PAH-Cd cocontaminated dumpsite soil by altering alfalfa growth and rhizosphere communities," *Science of The Total Environment*, vol. 806, p. 150610, Feb. 2022, doi: 10.1016/j.scitotenv.2021.150610.

[141] S. Shariati *et al.*, "Degradation of dibutyl phthalate by Paenarthrobacter sp. Shss isolated from Saravan landfill, Hyrcanian Forests, Iran," *Biodegradation*, vol. 33, no. 1, pp. 59–70, Feb. 2022, doi: 10.1007/s10532-021-09966-7.

[142] A. Kumar Awasthi, Z. Yuan, M. Kumar Awasthi, M. Li, S. Mishra, and A. Kumar Pandey, "Bioprocess potential of Eco-friendly fungal isolates converting organic waste to

bioresource,” *Bioresour Technol*, vol. 346, p. 126586, Feb. 2022, doi: 10.1016/j.biortech.2021.126586.

[143] L. A. Macías-Pérez *et al.*, “Contrasted microbial community colonization of a bauxite residue deposit marked by a complex geochemical context,” *J Hazard Mater*, vol. 424, p. 127470, Feb. 2022, doi: 10.1016/j.jhazmat.2021.127470.

[144] B. Choi, J. S. Yu, G. Y. Kang, T. Y. Jeong, E. H. Jho, and S. J. Lee, “Pyrolytic Remediation and Ecotoxicity Assessment of Fuel-Oil-Contaminated Soil,” *Toxics*, vol. 10, no. 5, May 2022, doi: 10.3390/toxics10050245.

[145] “Bioleaching of As from mine tailings using an autochthonous Bacillus cereus strain”.

[146] P. Perczyk and M. Broniatowski, “Membrane composition and successful bioaugmentation. Studies of the interactions of model thylakoid and plasma cyanobacterial and bacterial membranes with fungal membrane-lytic enzyme Lecitase ultra,” *Biochimica et Biophysica Acta (BBA) - Biomembranes*, vol. 1864, no. 6, p. 183888, Jun. 2022, doi: 10.1016/J.BBAMEM.2022.183888.

[147] A. Hassan *et al.*, “Bioaugmentation-assisted bioremediation and kinetics modelling of heavy metal-polluted landfill soil,” *International Journal of Environmental Science and Technology*, vol. 19, no. 7, pp. 6729–6754, Jul. 2022, doi: 10.1007/s13762-021-03626-2.

[148] Y. Ren, Z. Zhang, and M. Huang, “A review on settlement models of municipal solid waste landfills,” *Waste Management*, vol. 149, pp. 79–95, Jul. 2022, doi: 10.1016/j.wasman.2022.06.019.

[149] M. Martínez-Ruiz *et al.*, “Micro-algae assisted green bioremediation of water pollutants rich leachate and source products recovery,” *Environmental Pollution*, vol. 306, p. 119422, Aug. 2022, doi: 10.1016/J.ENVPOL.2022.119422.

[150] P. Sharma *et al.*, “A critical review on microbes-based treatment strategies for mitigation of toxic pollutants,” *Science of The Total Environment*, vol. 834, p. 155444, Aug. 2022, doi: 10.1016/j.scitotenv.2022.155444.

[151] J. Espada, R. Rodríguez, V. Gari, P. Salcedo-Abraira, and L. Fernando Bautista, “Coupling phytoremediation of Pb-contaminated soil and biomass energy production: A comparative Life Cycle Assessment”, doi: 10.1016/j.scitotenv.2022.156675ï.

[152] A. Bhagwat, C. S. P. Ojha, A. Pant, and R. Kumar, “Interaction among Heavy Metals in Landfill Leachate and Their Effect on the Phytoremediation Process of Indian Marigold,” *J Hazard Toxic Radioact Waste*, vol. 27, no. 1, Jan. 2023, doi: 10.1061/(ASCE)HZ.2153-5515.0000731.
